# Efficacy of a 3 month training program on the jump-landing technique in jump-landing sports. Design of a cluster randomized controlled trial

**DOI:** 10.1186/1471-2474-11-281

**Published:** 2010-12-13

**Authors:** Inne Aerts, Elke Cumps, Evert Verhagen, Romain Meeusen

**Affiliations:** 1 Vrije Universiteit Brussel, Faculty of Physical Education and Physical Therapy, Department of Human Physiology and Sports Medicine, Brussels, Belgium; 2 EMGO Institute for Health and Care Research, Department of Public and Occupational Health. VU University Medical Center, Amsterdam, the Netherlands

## Abstract

**Background:**

With the relatively high rate of injuries to the lower extremity due to jump-landing movement patterns and the accompanied high costs, there is need for determining potential preventive programs. A program on the intervention of jump-landing technique is possibly an important preventative measure since it appeared to reduce the incidence of lower extremity injuries. In real life situations, amateur sports lack the infrastructure and funds to have a sports physician or therapist permanently supervising such a program. Therefore the current prevention program is designed so that it could be implemented by coaches alone.

**Objective:**

The objective of this randomized controlled trial is to evaluate the effect of a coach supervised intervention program targeting jump-landing technique on the incidence of lower extremity injuries.

**Methods:**

Of the 110 Flemish teams of the elite division, 24 teams are included and equally randomized to two study groups. An equal selection of female and male teams with allocation to intervention and control group is obtained. The program is a modification of other prevention programs previously proven to be effective. All exercises in the current program are adjusted so that a more progressive development in the exercise is presented. Both the control and intervention group continue with their normal training routine, while the intervention group carries out the program on jump-landing technique. The full intervention program has a duration of three months and is performed 2 times a week during warm-up (5-10 min). Injuries are registered during the entire season.

**Discussion:**

The results of this study can give valuable information on the effect of a coach supervised intervention program on jump-landing technique and injury occurrence. Results will become available in 2011.

**Trial registration:**

Trial registration number: NTR2560

## Background

Different studies have pointed out that injuries in jump-landing sports are of frequent occurrence [1-4]. In volleyball, gymnastics and basketball respectively, a total of 2.8 [CI 95%: 2.0 -3.6], 2.2 [CI 95%: 1.6 - 2.9] and 9.8 [CI 95%: 8.5 - 11.1] injuries per 1,000 exposure hours have been reported [5,6]. The majority of the injuries in these sports occur at the lower extremity, accounting for more than 60% of all injuries [2-4]. Both overuse (e.g. shin splints, stress fractures [7], patellar tendinopathy (PT) [8,9], patellar femoral pain syndrome (PFPS) [10,11] and acute injuries (e.g. ACL injury [12-14] and ankle sprains [15-17]) often occur in jump-landing sports.

Sports injuries often are accompanied by pain or other physical discomforts, which in turn result in use of healthcare resources and possibly also in absenteeism from work. The severity of sports injuries can be described based on different criteria including sporting time lost, permanent damage and cost [6]. In a study of Cumps et al. (2007) the number of basketball days for which the athlete was not able to play or train because of the injury sustained was set on 3 to 7 weeks [18]. This means that injury occurrence can put a stop to the athlete's participation in his sport for a long time. From the study of Cumps et al. (2008)[6] it also can be concluded that the direct medical and the indirect costs in Flanders involved in acute injuries only, are respectively 15,027,423 € and 111,420,813 €. No information of the cost of overuse injuries was found, so it can be assumed that the actual cost of both acute and overuse injuries actually is much higher. Even more, important aspects of injury occurrence are the long term consequences. After, for example an ACL-injury or episodes of anterior knee pain, many athletes seem predisposed to osteoarthritis [19-21]. This can already occur at an early age and result in lifelong constraint in sport participation [19,20]. In order to reduce short and long term consequences, there is a need for sports injury prevention.

Arguably the most important injury mechanism for these lower extremity acute and overuse injuries is the jump-landing maneuver [1,3,5]. Proper jump-landing movement patterns are essential to absorb the generated impact forces efficiently, and are suggested to be strongly related to the athlete's risk for injuries to the lower extremity [22,23]. Consequently, various jump-landing related risk factors, such as increased knee valgus during jump-landing technique, ankle instability or a stiff landing technique, appear to be related with injury occurrence [8,24-26]. For example, a stiff landing strategy increases the risk of sustaining injuries due to higher ground reaction forces and decreased shock absorption during landing. Specific movement patterns such as dynamic valgus or stiff jump-landings technique can be a risk factor for respectively ACL injuries and PT [8,24,26,27].

With the relatively high rate of injuries of the lower extremity in jump-landing sports and the severity of these injuries, there is a need for practical preventive programs [28,29]. A program aiming at an improvement of jump-landing technique is potentially an important preventive measure while it is able to target a variety of risk factors for a variety of injuries. Previous attempts have been made to prevent specific injuries through improvement of jump-landing technique, e.g. recurrent ankle sprains [30] and ACL injuries [24]. However, most available prevention programs were supervised by sport physicians or physical therapists. This might work for (sub)elite sports were medical supervision is always available, but amateur sports lack the infrastructure and funds to have a sports physician or therapist permanently supervising such a program. Nevertheless, it is amateur sports where, in absolute sense, the injury burden is highest through high participation numbers. Therefore, the true effectiveness of available preventive programs in this important setting is questionable.

### Objective

The objective of this randomized controlled trial is to evaluate the effectiveness of a coach supervised intervention program targeting jump-landing technique on the incidence of lower extremity injuries.

### Research questions

- What is the effectiveness of a coach supervised jump landing intervention program on the incidence of acute and overuse injuries to the lower extremity?

- What is the effect of a coach supervised jump landing intervention program on jump landing technique of individual athletes?

## Methods

The CONSORT statement was followed to describe the design of this study. This statement is a checklist intended to improve the quality of reports of randomized controlled trials [31].

### Study outline

A two-way cluster randomized controlled trial with a follow-up period of one season (6 months) is used. The participation subjects are athletes from basketball teams of the national divisional, and, 1^st ^and 2^nd ^regional divisional basketball competitions.

The study protocol is accepted by the local ethical committee of the Free University Brussels (B.U.N. B14320071963) and a trial registration number was requested (NTR2560).

This study is financially supported by the Flemish Government through the establishment of the Policy Research Center Sports, Youth and Culture.

### Participants

Athletes who are actively participating in the national division and, 1^st ^and 2^nd ^regional basketball teams in Flanders, Belgium, are eligible for inclusion in the study. Athletes are excluded if they do not master the Dutch language or have a current injury to the lower extremity at time of inclusion.

Written informed consent is obtained from each athlete and the study is conducted in accordance with the ethical institutional rules for human research and in regulation with the Declaration of Helsinki for Medical Research involving human subjects.

### Sample size

A power calculation is carried out for the main outcome variable lower extremity injury incidence. A difference of 50% in the incidence of lower extremity injuries between the intervention and control group after a follow up of one season is considered to be clinically relevant.

The prevalence of lower extremity injuries in basketball in Flanders being about 78% in one season [6], 34 subjects per group are needed to detect the intended difference of 50% in the incidence of lower extremity injuries, with a power of 90% and an alpha of 5%. Assuming a dropout rate of about 20% a total of 82 athletes are needed to detect a potentially clinically relevant effect of the intervention. However, as teams serve as unit of randomization, a cluster effect should be taken into account. Therefore, an intra-cluster correlation coefficient of 20% was considered, resulting in a total of 240 athletes from 24 teams is necessary at baseline.

### Recruitment

**Figure 1:**
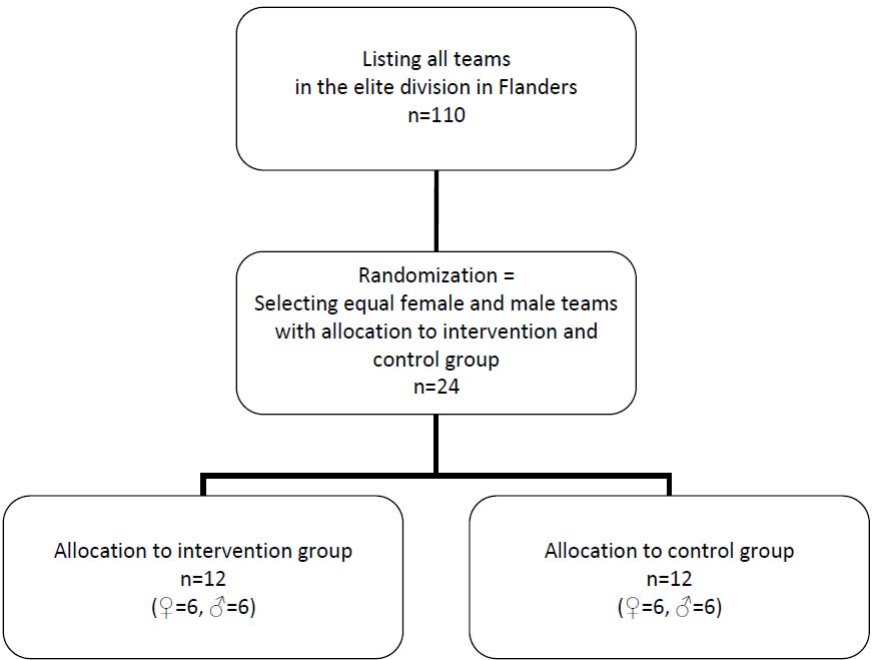
**Flowchart recruitment**.

Of the 110 Flemish teams of the elite division, 24 teams are included and equally randomized to two study groups (Figure 1). Randomization and group allocation takes place before teams were contacted. This is done in order to avoid any spill over of the intervention while including teams. Through this method blinding of the teams to group allocation is secured.

### Intervention program

After receiving informed consent and baseline questionnaire of a team's individual athletes, trainers are provided with a package that enclosed the necessary information. Control gorup (CG) trainers are provided with a DVD containing general information on the study. Trainers of the intervention teams receive a different DVD with general information on the study and specific information on the intervention program and the individual exercises. Also a written manual including the complete program and a detailed explanation for each individual exercise is provided.

Both research groups continue their regular training routine. Teams of the intervention group (IG) receive an additional intervention program to be carried out during the regular warm-up (Table 1). This program targets at improving the athletes' jump-landing technique.

**Table 1:** Overview of the jump-landing technique program

2 ×/week, **10 min**. 1 min rest between repetitions	Technique/ Month 1			Fundamentals/ Month 2			Performance/ Month 3		
Week 1	Co-contractions	10	L + R	Lying position	15		X-Hops	6	Cycles L + R
	Wall Squat	10		Pelvic bridge	10	sec	Hop-Hop-Hold	8	L + R
	Lateral Jump and Hold	8	L + R	Repeated Tuck Jumps	10	L + R	Mattress jumps	30	sec
	Front Lunges	10		Squat Jump	10		Single leg 90°*	8	L + R
	Step-Hold	8	L + R	Jump Single Leg Hold	8		Max Squat jumps-hold	10	L + R

Week 2	Co-contractions	10	L + R	Pelvic Bridge Single Leg	10		Crossover-Hop-Hop-Hold	8	L + R
	Squat	10		Prone Bridge Hip (EK) Shoulder Flexion	10	L + R	Single Leg 4 Way Hop-Hold*	3	Cycles L + R
	Step-Hold	8	L + R	Side to Side Tuck Jump	10	sec	Single leg 90° Ball*	8	L + R
	Walking Lunges	10		Lateral Hop & Hold	8	L + R	Step, jump up, down, vertical jump	5	L + R
	Lateral Jump and Hold	8	L + R	Hop & Hold	8		Max Squat jumps-hold	10	

Week 3	Squat	10		Single Leg Pelvic Bridge*	10	L + R	Single Leg 4 Way Hop-Hold Ball*	4	Cycles L + R
	Lateral Jump and Hold	8	L + R	Prone Bridge Hip extension	10	L + R	Single Leg 180°	10	L + R
	Single Tuck Jump Soft Landing	10	L + R	Side to Side Tuck Jumps	10	L + R	Jump, Jump, Jump, vertical jump	10	sec
	Lunge Jumps	10		Lateral Hops	10	sec	Mattress jumps	40	L + R
	Lateral Jumps	10	sec	Double leg 90°	8	L + R	Running, Jump down 1 legged, Jump	8	

Week 4	Squat Jumps	10		Single Leg Pelvic Bridge Ball	10	L + R	Single Leg 180°	10	L + R
	Lateral Jumps	10	sec	Prone Bridge Hip Opposed Shoulder Flexion	10	L + R	Jump, Jump, Jump, vertical jump	15	L + R
	Double Tuck Jump	8		Lateral Hops with ball	10	sec	Running, Jump down 1 legged, Jump	10	
	Broad Jump	10	L + R	Single Leg Lateral Hop-Hold	5	L + R	Lay-up ^	10	
	Scissor Jumps	8		Single leg 90°	8	L + R	Height jump ^	10	

*Exercise on the mattress

^ Sport specific jumps for basketball, can be adjusted depending on the sport.

The program is a modification of two prevention programs previously proven to be effective for the prevention of lower extremity injuries [24,32]. The exercises in the current program are adapted so that a more progressive development is present. Furthermore, the materials required to complete the exercises are limited. The program has a duration of three months and is performed twice a week during warm-up (5-10 min). All exercises are selected so that the jump-landing program difficulty is gradually increased. First, basic techniques are practiced and then fundamental exercises are trained. Hereafter, more difficult and sport specific exercises are given in the performance phase. The intervention is considered to be appropriate for all athletes and to have no negative side-effects.

### Measurements

During the study period there are two fixed measurements, at baseline (T0) and after 3 months (T1). At T0 a questionnaire is completed by the athletes, and at both occasions the athletes' jump landing technique is assessed. Additionally, exposure hours and lower extremity injuries are continuously registered during the entire 2010-2011 basketball season (6 months).

#### Questionnaire

At T0 all athletes complete a questionnaire on demographic data, previous injuries, sports history and current sports participation [33].

#### Jump landing technique

An improvement in jump-landing technique is determined by registering jump-landing technique of athletes in the CG and IG. Athletes are asked to perform 3 separate maximal vertical jumps which are recorded on camera (SONY HDV 1080i). The researchers collect the jump-landing data at T0 and T1. These jumps are all analyzed through the computer program Darttrainer^®^. In addition, all jumps are evaluated through a predefined list of criteria, the JLS-System (Jump-landing Scorings system) (Table 2). This tool is developed as a field test to evaluate proper jump-landing technique.

**Table 2:** Scorings system Jump-landing Technique (JLS-system)

Front view (observers view)				
***Criteria***	***Description***	***Observation***	***Measurement***	***Score***

Maintaining 'balance' *- Loss of balance*	Lifting one foot or toes of both feet due to falling.	Observe complete jump	Loss of balance present or not	Loss of balance not present or doubt: 0 Loss of balance present: 1
*- Centre of gravity displacement*		At first foot contact: Perpendicular through incisura jugularis	At maximal knee flexion determine the position of incisura jugularis (left/right) compared to perpendicular	Displacement not present or doubt: 0 Displacement left or right: 1

Landing with '*both feet at the same time'*	Not simultaneous: obviously 2 contact times of the feet visible	Observe complete jump	Simultaneous landing present or not	Simultaneous landing present or doubt: 0 Simultaneous landing not present: 1

*'Muscular control' *during landing	Presence of eccentric control during landing, capacity to lower the body in a controlled manner (no side movements of the knee, no sudden stops)	Observe complete jump	Muscular control present or not	Muscular control present or doubt: 0 Muscular control not present: 1

*'Genu varum' - 'Genu valgum' take-off*	Malalignement: valgus or varus position	At maximal knee flexion: perpendicular from SIAS to ankle (middle of malleoli) - Take-off	No varum or valgum: perpendicular goes through centre of patella	Perpendicular through patella or doubt: 0 Patella lateral from the perpendicular: (1 = varum) Patella medial from the perpendicular: (2 = valgum)

*'Genu varum' - 'Genu valgum' landing*	Malalignement: valgus or varus position	At maximal knee flexion: perpendicular from SIAS to ankle (middle of malleoli) - Landing	No varum or valgum: perpendicular goes through centre of patella	Perpendicular through patella or doubt: 0 Patella lateral from the perpendicular: (1 = varum) Patella medial from the perpendicular: (2 = valgum)

*'Position of feet'*	Feet at shoulder width.	At maximal knee flexion: Perpendicular trough centre of shoulders.	Perpendicular goes trough centre of malleolus internus and externus	Perpendicular through centre of malleoli or doubt: 0 Perpendicular not through centre of malleoli: 1

*'Distance between knees*' compared between initial contact and maximal knee flexion	Distance between knees at maximal knee flexion, 80-110% of distance between knees at initial contact.	Distance between knees at first foot contact and distance between knees at maximal knee flexion.	Range of 80%-110% difference.	Between 80%-11%: 0 Not between 80%-110%: 1

*Hyperpronation - Hypersupination feet* *Take-off*	Inside or outside movement of the malleoli compared to starting position.	Observe complete jump - Takeoff	Compare position feet with starting position.	Hyperpronation or -supination not present or doubt: 0 Hyperpronation or supination present: 1

*Hyperpronation - Hypersupination feet* *landing*	Inside or outside movement of the malleoli compared to starting position.	Observe complete jump - Landing	Compare position feet with starting position.	Hyperpronation or -supination not present or doubt: 0 Hyperpronation or supination present: 1

**Side view** **(right and left)**				

***Criteria***	***Description***	***Observation***	***Measurement***	***Score***

Knee angle *- First foot contact* *- Maximal knee flexion*		Knee angle at first foot contact Knee angle at maximal knee flexion	Ankle between trochanter major - caput fibulae (tractus iliotibialis) - mallolus externus	Degrees:

Hip angle *- First foot contact* *- Maximal knee flexion*		Knee angle at first foot contact Knee angle at maximal knee flexion	Angle between trunk - trochanter major - caput fibulae (tractus iliotibialis)	Degrees:

Ankle angle *- First foot contact* - *Maximal knee flexion*		Knee angle at first foot contact Knee angle at maximal knee flexion	Angle between caput fibulae (tractus iliotibialis) - malleolus externus - parallel with sole of foot	Degrees:

*'shoulders above knees'*		Position at maximal knee flexion	Perpendicular through centre of shoulder (humerus head)	Perpendicular through caput fibula: 0 Perpendicular not through caput fibula: 1

*'knees not further than toes'*		Position at maximal knee flexion	Perpendicular through centre of knees	Perpendicular not further than toes: 0 Perpendicular further than toes: 1

*'forefoot landing and rolling to heel'*	Forefoot landing and rolling to heel	Observe complete jump	Pattern present or not	Pattern present or doubt: 0 Pattern not present: 1

*Arm swing*	Obvious arm swing that contributes to the jump height	Observe complete jump	Arm swing present or not	Arm swing present: 0 Arm swing not present: 1

#### Exposure time

Exposure is recorded by the coach on an exposure form. Coaches note the total duration of each training session and match, and classify the level of participation of each player (that is, in terms of full, three quarters, one half, one quarter, or no participation). If the player does not participate fully, the coach note the reason--that is, being injured, ill, or absent for other reasons. Completed exposure forms are returned on a weekly basis. Every week trainers are contacted by phone and urged to fill out the exposure sheets, and, if necessary, the injury forms. In this, we followed the example of Verhagen et al.[2] who applied the same method of exposure registration.

#### Injury definition

To determine the effectiveness of the program for the prevention of injuries, all injuries that occur during the entire season are registered in both the IG and CG. Injuries are divided into two main categories: acute or overuse injuries. An acute injury is defined as a sports accident, with a sudden, direct cause/onset, which requires at least minimal (medical) care (e.g. ice or taping) and which causes the injured subject to miss out on at least 1 training session. Exclusion criteria involve muscle cramps and mild bruises. A subject sustains an overuse injury if he or she experiences a physical discomfort, with an insidious onset, that caused pain and/or stiffness of the musculoskeletal system, and that is present before, during and/or after the sports activity. The overuse injury is only registered if present for at least three consecutive sessions. Illness and fatigue are not registered as overuse injuries [18].

#### Injury registration

Coaches receive injury forms at the start of the study. In case of injury, the coach completes in concordance with the injured player injury registration forms, which have to be completed within one week after injury onset. On this form the player is asked to provide information on the injury location, injury type, diagnosis of the injury, direct cause of the injury, preventive measures used at the time of the injury, first aid given, and subsequent medical treatment. If an injury is noted on the exposure form and no injury registration form is received within two weeks after the injury is logged, the coach is contacted and urged to let the player complete the injury registration form.

### Process evaluation & Compliance

Coaches are contacted on a weekly basis by means of a telephone call. The goal of this call is to inquire about the ongoing of the study and to follow-up on any missing exposure or injury registration forms. In addition, IG coaches are asked whether they followed the prescribed exercises for that particular week. If not, the exercises that are carried are noted in order to gain insight into the compliance to the program.

At T1 coaches are required to complete a questionnaire inquiring on their opinion of the intervention program, their compliance to the prescribed exercises, and to inquire about any difficulties in executing the program.

### Statistical analyses

The injury incidence density (IID) is calculated as the number of new injuries per 1,000 hours of participation, using exposure time of each individual participant until the first injury. The number of injuries divided by the total time at risk is the preferred measure of incidence because it can accommodate variations in the exposure time of individuals [34,35]. If an athlete suffers multiple injuries, only the first injury will be considered in the analysis.

Because the unit of allocation was teams, we perform a multilevel Cox proportional hazard regression analysis, using the computer based software SPSS 17.0 (SPSS Inc, Chicago, Illinois, USA), to estimate the hazard ratios (HRs) and 95% confidence intervals (CIs). Teams will be used as cluster levels [36].

The difference between the CG and IG regarding the JLS-system is calculated using Repeated Measures ANOVA (p < 0.05) for normally distributed data and the Mann-Whitney-*U *test (p < 0.05) for data which are not normally distributed.

### Implications of this study

This study evaluates the effect of a coach supervised injury prevention program. If successful this will provide valuable information for sports injury prevention practice. As the majority of injuries are sustained in amateur sports where medical guidance is limited, effective coach supervised prevention has a great potential impact on public health.

## Competing interests

The authors declare that they have no competing interests.

## Authors' contributions

IA, EC, EV, RM were involved in the conception and design of the study. IA is involved in study data collection. All authors were involved in drafting the manuscript and revising it for critically important content. All authors have read and approved the final manuscript.

## Pre-publication history

The pre-publication history for this paper can be accessed here:

http://www.biomedcentral.com/1471-2474/11/281/prepub
